# Comparing Multiple Imputation Methods to Address Missing Patient Demographics in Immunization Information Systems: Retrospective Cohort Study

**DOI:** 10.2196/73916

**Published:** 2025-08-26

**Authors:** Sara Brown, Ousswa Kudia, Kaye Kleine, Bryndan Kidd, Robert Wines, Nathanael Meckes

**Affiliations:** 1 Scientific Services - Analytics, Scientific Technologies Corporation (United States), 411 S 1st St, Phoenix, AZ, 85004, United States, 1 480-745-8500; 2 Immunization Services, West Virginia Department of Health and Human Services, Charleston, WV, United States

**Keywords:** multiple imputation, missing data, imputation methods, data science, machine learning, statistical modeling, immunization information system

## Abstract

**Background:**

Immunization Information Systems (IIS) and surveillance data are essential for public health interventions and programming; however, missing data are often a challenge, potentially introducing bias and impacting the accuracy of vaccine coverage assessments, particularly in addressing disparities.

**Objective:**

This study aimed to evaluate the performance of 3 multiple imputation methods, Stata’s (StataCorp LLC) multiple imputation using chained equations (MICE), scikit-learn’s Iterative-Imputer, and Python’s miceforest package, in managing missing race and ethnicity data in large-scale surveillance datasets. We compared these methodologies in their ability to preserve demographic distribution, computational efficiency, and performed G-tests on contingency tables to obtain likelihood ratio statistics to assess the association between race and ethnicity and flu vaccination status.

**Methods:**

In this retrospective cohort study, we analyzed 2021‐2022 flu vaccination and demographic data from the West Virginia Immunization Information System (N=2,302,036), where race (15%) and ethnicity (34%) were missing. MICE, Iterative Imputer, and miceforest were used to impute missing variables, generating 15 datasets each. Computational efficiency, demographic distribution preservation, and spatial clustering patterns were assessed using G-statistics.

**Results:**

After imputation, an additional 780,339 observations were obtained compared with complete case analysis. All imputation methods exhibited significant spatial clustering for race imputation (G-statistics: MICE=26,452.7, Iterative-Imputer=128,280.3, Miceforest=26,891.5; *P*<.001), while ethnicity imputation showed variable clustering patterns (G-statistics: MICE=1142.2, Iterative-Imputer=1.7, Miceforest=2185.0; *P*: MICE<.001, Iterative-Imputer=1.7, Miceforest<.001). MICE and miceforest best preserved the proportional distribution of demographics. Computational efficiency varied, with MICE requiring 14 hours, Iterative Imputer 2 minutes, and miceforest 10 minutes for 15 imputations. Postimputation estimates indicated a 0.87%‐18% reduction in stratified flu vaccination coverage rates. Overall estimated flu vaccination rates decreased from 26% to 19% after imputations.

**Conclusions:**

Both MICE and Miceforest offer flexible and reliable approaches for imputing missing demographic data while mitigating bias compared with Iterative-Imputer. Our results also highlight that the imputation method can profoundly affect research findings. Though MICE and Miceforest had better effect sizes and reliability, MICE was much more computationally and time-expensive, limiting its use in large, surveillance datasets. Miceforest can use cloud-based computing, which further enhances efficiency by offloading resource-intensive tasks, enabling parallel execution, and minimizing processing delays. The significant decrease in vaccination coverage estimates validates how incomplete or missing data can eclipse real disparities. Our findings support regular application of imputation methods in immunization surveillance to improve health equity evaluations and shape targeted public health interventions and programming.

## Introduction

The usage of large datasets obtained from surveillance data and Immunization Information Systems (IIS) has held a vital role in recognizing and comprehending the extent of health disparities and inequities within a population [1,2]. Previous research has shown that there is an association between race and ethnicity and vaccine acceptance and uptake [3-5]. However, as exemplified by the COVID-19 pandemic, race and ethnicity fields in public health surveillance systems and health records are historically underpopulated, thereby limiting a full understanding of the extent of vaccine inequities [2,6,7]. Missing data can affect the capability to effectively describe vaccine coverage and may introduce bias into epidemiologic analyses, particularly when attempting to estimate and address racial and ethnic health inequities, further compromising public health decision-making and resource allocation.

Studies exploring racial and ethnic inequalities traditionally omit individuals with missing demographic information or classify them as “unknown” [8-11]. However, this can miscalculate vaccine coverage rates when stratified by race and ethnicity, leading to biased analyses between different racial and ethnic groups. This selection bias dampens the capacity to accurately measure the true vaccine uptake among underserved populations that are more likely to have racial and ethnic data missing in surveillance datasets [2,12-14]. Multiple imputation for missing data, alternatively, has been shown to be a better method for eliminating missing data and preserving the greatest amount of data [13]. The theoretical foundation and technique for multiple imputation was established by D B Rubin, who developed the structure for managing missing data through the formation of multiple probable datasets [15]. This central process undertakes the innate ambiguity of missing values by generating multiple, complete, and reasonable datasets [16-18].

Following multiple imputation, classic likelihood ratio tests cannot be implemented as is because the final estimates do not come directly from a single model, and as a result, require modification to account for the uncertainty introduced by the imputation process. The multiple imputation likelihood ratio test, developed by Li et al [19] and Meng et al [20], or stacked multiple imputation, introduced by Chan et al [21,22], provides frameworks for hypothesis testing that properly incorporate both within-imputation and between-imputation variability.

Multiple imputations using chained equations (MICE) builds upon Rubin’s initial work to handle multifaceted and specific datasets; it is a statistically sound method for managing missing data and has been used in the context of medical research and large, national datasets [23,24]. MICE uses the distribution of the original data to estimate values that signify the ambiguity of the true missing value. This can yield unbiased approximations after an adequate number of imputations, which is contingent on the quality of the dataset.

Machine learning (ML) is increasingly being used to reconcile missing data by offering robust and sophisticated solutions for improving data quality. Different ML techniques use various mathematical processes based on decision trees to predict a specified outcome, all with varying levels of accuracy [25-28]. Similar to traditional MICE, the accuracy and performance of machine learning algorithms to impute missing data rely heavily on the type of missing data being evaluated.

When compared with various ML techniques, MICE had less bias and similar standard errors for parameter estimates, with additional studies demonstrating that combining MICE with ML yields less biased results [26]. Miceforest is a combination of classical epidemiological techniques and ML algorithms. This algorithm uses MICE with light gradient boosting, a tree-based algorithm, to provide a flexible and powerful solution that remains efficient and reliable for managing missing data [29-31].

Recent public health pandemics and outbreaks have emphasized the importance of reliable approaches to missing data in surveillance systems. Studies have demonstrated that imputation methods significantly affect results, with incidence estimates and measures of disparity varying by method, and multiple imputation demonstrating more consistency and efficacy than single or complete case approaches [2,32,33].

Recent attempts have explored Bayesian Surname and Geocoding and Bayesian Improved Surname and Geocoding (BISG) methods to reconcile missing race and ethnicity data in large public health datasets [2,34-36]. While this approach has demonstrated practicality in particular circumstances, its suitability for state-level immunization registries is imperfect. Specifically, name-based imputations can present systematic bias in racially and ethnically diverse populations [37]. Statewide immunization registries contain various naming conventions and regional uniqueness; reliance on a name-based imputation can bolster misclassification bias and lead to misguided epidemiological analyses. As a result, approaches such as multiple imputation using observed covariates may be more suitable for IISs [38-40].

IISs present unique missing data challenges because they are a birth-to-death registry, making them temporally complex. Missing data patterns may vary by birth cohort due to evolving reporting requirements, provider participant rates, state-specific reporting requirements, use of multiple providers, and technology advancements.

However, this research addresses a critical gap identified in the surveillance literature, where, despite the widespread recognition of missing data challenges, few studies have systematically evaluated imputation approaches specifically designed for immunization data. To date, there is little published peer-reviewed literature on methods to address missing data in IIS. A 2024 cross-sectional study improved race and ethnicity data in the NYS laboratory reporting system by integrating IIS data, while Russ et al [41] enhanced IIS completeness by incorporating external records and applying logistic multiple imputation and random forest techniques [42].

This study builds upon established multiple imputation methodologies while addressing the complexities of IIS data structures by adapting recent advancements in handling missing data in EHRs to the specific challenges of population-based immunization surveillance. In this study, we investigate racial and ethnic disparities in flu vaccine uptake in the West Virginia IIS for the 2021‐2022 flu season. We sought to address potential bias due to missing race and ethnicity data through multiple imputation and test the efficiency and accuracy of 3 established methods to address missing data in IIS and the implications of imputations on flu vaccination coverage data.

## Methods

### Overview

In this retrospective cohort, patient data for the 2021‐2022 flu vaccination (June 1, 2021, to June 30, 2022) was obtained from the West Virginia Statewide Immunization Information System (WVSIIS). Geo-demographic data, such as urbanicity and Social Vulnerability Index (SVI) status, were calculated using county address data obtained from the IIS. For the purposes of our analysis, urbanicity was defined as a county being either “metropolitan” or “rural.” In accordance with the Federal Office of Rural Health Policy (FORHP), counties that had a population of 50,000 or more were considered “metropolitan” and counties with less than 50,000 were considered “rural.” County population was obtained from the West Virginia 2020 US Census [43,44]. SVI is calculated by the Centers for Disease Control and Prevention (CDC) and the Agency for Toxic Substances and Disease Registry (ATSDR) at the census tract and county level and represents the potential negative effects of external stresses, such as housing, socioeconomic, and minority statuses have on a community [45].

After a preliminary data cleaning process on a clone of the WVSIIS database, where patients who were marked as deceased, inactive, or retained out of state address were removed, there were a resulting 2,302,036 records with a 2021‐2022 flu vaccine coverage rate of 26%. 15% of records were missing patient race (n=347,633), and 34% (n=780,339) of records were missing patient ethnicity. We addressed missing race and ethnicity data by using 3 different multiple imputation approaches to assess the stability and reliability of our findings.

We first implemented Stata 17’s (StataCorp LLC) MICE using the mi impute chained command. Stata allows us to identify individual imputed datasets and pool results, excluding the original dataset with missing values.

We then used the Python scikit-learn’s Iterative-Imputer algorithm. Iterative Imputer operates similarly to MICE by using available data in other features to estimate missing values. The imputation is performed in an iterative, round-robin manner, with a regressor to predict the missing values [46].

We applied the Python Miceforest package, which is designed for performing MICE using random forests. Rather than assume a linear relationship between variables, miceforest can capture complex, non-linear patterns and imputes values iteratively, unlike traditional MICE [33].

With each imputation method, we created 15 imputed datasets to stabilize our variance estimates [47,48]. We jointly imputed patient race and ethnicity using age, sex, urbanicity, SVI, county, and flu vaccination status. Selection of these covariates was based on the work done by Zhang et al [40], and the considerable missingness present in other potentially informative variables. Once missing data had been reconciled, postimputation estimates were applied to WVSIIS flu vaccination data for the 2021‐2022 flu season.

We performed G-tests on contingency tables to obtain likelihood ratio statistics to assess the association between race and ethnicity and flu vaccination status. For MICE, we pooled across individual imputed datasets, and G-test statistics were averaged across imputations. In addition, we calculated between-imputation variance to evaluate the stability of results. Pooling was used in lieu of stacking to accommodate the computational requirements of the statewide immunization data’s size. No pooling was required of Iterative-Imputer and Miceforest because they produce a single imputation result.

To assess the proportion estimate, we calculated vaccination proportions with 95% CIs using the Wilson score interval method. This method offers improved coverage properties than the standard normal estimate.

Sensitivity analyses examined vaccination disparity statistical significance, consistency, comparison of G-statistics to determine variances in effect sizes, and whether CIs for vaccination proportions overlap.

The primary engine for Iterative-Imputer, Miceforest, and all postimputation analyses was executed in Python 3.11 using pandas, scipy.stats, and matplotlib. The evaluation framework was intended to manage the multilevel configuration of Stata’s MICE while preserving uniformity with Iterative-Imputer and Miceforest. All Python computations were performed on a cloud-based computing cluster with 16-core processors and 128 GB of RAM.

### Ethical Considerations

This study consisted of secondary analysis of deidentified data from WVSIIS. The original data collection was conducted as part of state-mandated public health surveillance under W Va Code R § 64-7-6.6, which requires immunization data collection for all individuals residing in West Virginia. As such, it was not subject to institutional review board (IRB) review [49]. The secondary analysis did not involve direct interaction with human candidates and was determined not to constitute human candidate research, qualifying for exemption from IRB review and not requiring informed consent. This study met the jurisdiction requirement for IRB exemption, given the nature of the data used. The data used were compiled for public health surveillance and did not contain personal designations. Consent to access and analyze the data was permitted by the West Virginia Division of Immunization Services. All analyses met the terms of related federal, state, and international data privacy regulations. No compensation was provided or applicable, and no images or materials containing identifiable individual information are included in the manuscript or supplementary files.

## Results

### MICE

347,633 race categories that were previously missing were imputed after completing MICE. There were 16.5% additional White records, 16.8% additional Black records, 16.3% additional Asian records, 18.5% additional Indigenous records, 16.8% additional Native Hawaiian or Pacific Islander records, 17.4% additional Multiracial records, and 15.3% additional other records after imputations (Table 1).

780,339 ethnicity categories that were previously missing were imputed after completing MICE. There were 52.4% additional Hispanic or Latino records and 40.5% additional Not Hispanic or Latino records after imputations (Table 1).

**Figure 1.**
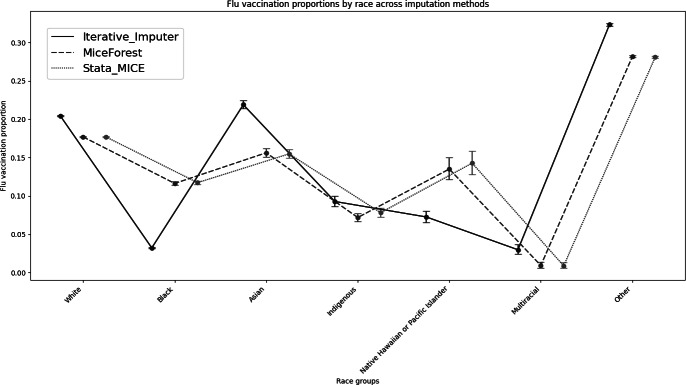
Flu vaccination coverage proportions with 95% CIs by race across multiple imputation using chained equations, Iterative-Imputer, and Miceforest of the West Virginia Statewide Immunization Information System flu data, 2021‐2022 (N=2,302,036).

After MICE, individual demographics remained proportional to the original dataset distributions (Figures1  2 and Table 2). A chi-square test illustrated that there was a statistically significant difference between the complete case and MICE estimates (*P*<.001; Table 3). Overall computational time was approximately 14 hours.

For the 2021‐2022 flu season, the complete case analysis flu vaccine coverage rate was 26%. After MICE, West Virginia had an overall flu vaccine coverage rate of 19%. Flu vaccine coverage rates decreased when stratified by race and ethnicity when compared with a complete case analysis, with the most significant decreases observed in non-Hispanic (NH) White (5%), NH Black (4%), Hispanic or Latino (6%), Asian (7%), Native Hawaiian or Pacific Islander (6%), and Other (19%; Table 4 and Figure 3). After reconciling missing race and ethnicity, an additional 63,984 individuals were included in the analysis that were previously excluded from stratified analyses.

Likelihood ratio tests were used to determine the impact of race and ethnicity on vaccine uptake. Using MICE, race was significantly associated with flu vaccination status (G-statistic range=26,365.427‐26,612.980; pooled G-statistic=26,452.66; *P*<.001), with considerable between-imputation variance (4103.38; Table 5). This denotes good stability with a coefficient of variation of 0.24%. For ethnicity, the MICE model also denoted a significant impact (G-statistic range=1055.287‐1,205.254; pooled G-statistic=1142.23; *P*<.001), also with substantial between-imputation variance (1277.885) and a moderate coefficient of variation of 3.13% (Table 5). Both of MICE’s G-scores demonstrate a large effect.

**Table 1.** Assessment of West Virginia Statewide Immunization Information System race and ethnicity demographics using complete case analysis and 3 imputation methods—multiple imputation using chained equations, Iterative-Imputer, and Miceforest.

Variable	Missing, %	Complete case		MICE^a^		Iterative-Imputer		Miceforest	
Race	15%								
White, n (%)		1,519,326 (77.7)		1,793,167 (77.8)		1,527,855 (66.4)		1,792,102 (77.8)	
Black, n (%)		65,716 (3.36)		77,767 (3.38)		391,756 (17.0)		78,624 (3.42)	
Asian, n (%)		14,334 (0.73)		16,886 (0.73)		24,003 (1.04)		16,813 (0.73)	
Indigenous, n (%)		7110 (0.36)		8561 (0.37)		7110 (0.31)		9399 (0.41)	
Native Hawaiian or Pacific Islander, n (%)		1720 (0.09)		2036 (0.09)		4846 (0.21)		2235 (0.10)	
Multiracial, n (%)		2405 (0.12)		2863 (0.12)		2674 (0.12)		2755 (0.11)	
Other, n (%)		343,792 (17.6)		400,757 (17.4)		343,792 (14.9)		400,108 (17.4)	
Total, N		1,954,403		2,302,036		2,302,036		2,302,036	
Ethnicity	34%								
Hispanic or Latino, n (%)		33,652 (2.21)		57,552 (2.50)		33,652 (98.5)		75,031 (3.26)	
Not Hispanic or Latino, n (%)		1,488,045 (97.8)		2,244,484 (97.5)		2,268,384 (1.46)		2,227,005 (96.7)	
Total, N		1,521,697		2,302,036		2,302,036		2,302,036	

a MICE: multiple imputation using chained equations.

**Figure 2.**
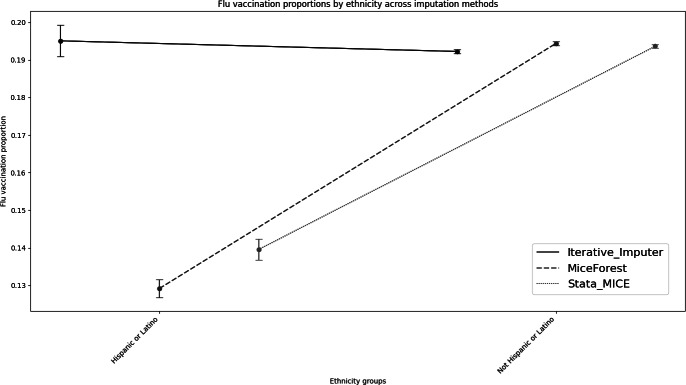
Flu vaccination coverage proportions with 95% CIs by ethnicity across multiple imputation using chained equations, Iterative-Imputer, and Miceforest of the West Virginia Statewide Immunization Information System flu data, 2021‐2022 (N=2,302,036).

**Table 2.** 2021‐2022 WVSIIS^a^ flu vaccination proportions with 95% CIs by race and ethnicity after multiple imputation using chained equations, Iterative-Imputer, and Miceforest.

Variables	MICE^b^ (95% CI)	Iterative-Imputer (95% CI)	Miceforest (95% CI)
Race
White	0.177 (0.176‐0.178)	0.205 (0.204‐0.205)	0.177 (0.176‐0.178)
Black	0.117 (0.115‐0.120)	0.032 (0.032‐0.033)	0.116 (0.114‐0.119)
Asian	0.155 (0.150‐0.160)	0.219 (0.214‐0.224)	0.156 (0.151‐0.162)
Indigenous	0.078 (0.073‐0.084)	0.093 (0.086‐0.100)	0.072 (0.067‐0.077)
Native Hawaiian or Pacific Islander	0.143 (0.128‐0.159)	0.073 (0.066‐0.080)	0.135 (0.122‐0.150)
Multiracial	0.009 (0.006‐0.013)	0.030 (0.024‐0.037)	0.009 (0.006‐0.014)
Other	0.281 (0.280‐0.282)	0.323 (0.322‐0.325)	0.282 (0.280‐0.283)
Ethnicity			
Hispanic or Latino	0.140 (0.137‐0.142)	0.195 (0.191‐0.199)	0.129 (0.127‐0.132)
Not Hispanic or Latino	0.194 (0.193‐0.194)	0.192 (0.192‐0.193)	0.194 (0.194‐0.195)

a WVSIIS: West Virginia Statewide Immunization Information System.

b MICE: multiple imputation using chained equations.

**Table 3.** Chi-square tests evaluating race and ethnicity distributions in the West Virginia Statewide Immunization Information System among the complete case dataset and the multiple imputation using chained equations-imputed, Iterative-Imputer-imputed, and Miceforest-imputed datasets.

Imputation method	Pearson chi-square	*P* value
MICE^a^	1.1e+04	<.001
Iterative-Imputer	1.8e+05	<.001
Miceforest	1.3e+04	<.001

a MICE: multiple imputation using chained equations.

**Figure 3.**
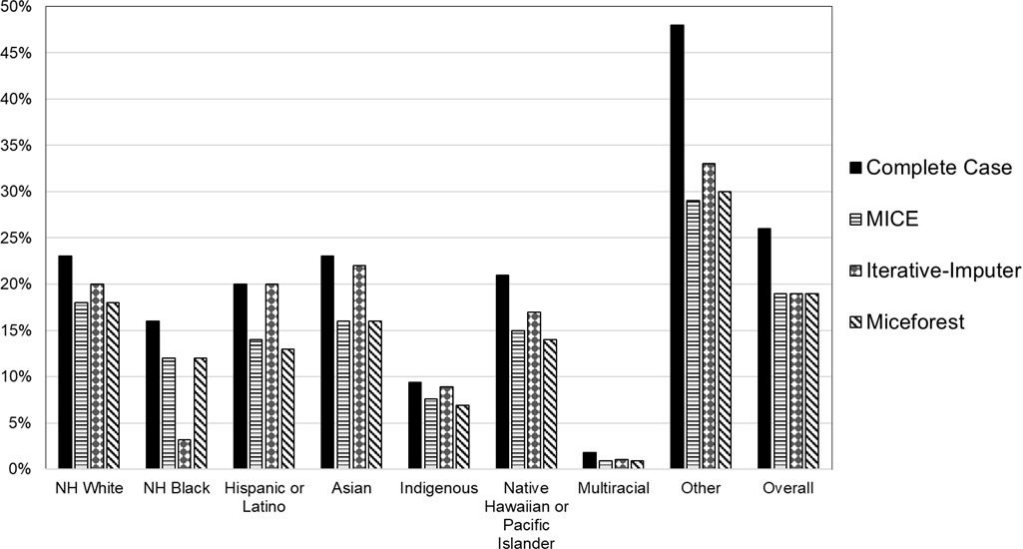
Flu vaccination coverage proportions by race and ethnicity across multiple imputation using chained equations, Iterative-Imputer, and Miceforest of the West Virginia Statewide Immunization Information System flu data compared against the complete case analysis, 2021‐2022 (N=2,302,036). NH Black: non-Hispanic Black; NH White: non=Hispanic White.

**Table 4.** 2021‐2022 West Virginia Statewide Immunization Information System flu vaccination counts for complete case, multiple imputation using chained equations, Iterative-Imputer, and Miceforest by race and ethnicity.

Race and ethnicity	Complete case (N=378,574), n (%)	MICE^a^ (N=442,679), n (%)	Iterative-Imputer (N=442,679), n (%)	Miceforest (N=442,679), n (%)
NH^b^ White	274,597 (72.5)	313,530 (71.1)	309,150 (69.8)	312,686 (70.6)
NH^b^ Black	7403 (1.96)	8931 (2.02)	12,510 (2.83)	8903 (2.01)
Hispanic or Latino	6565 (1.73)	8031 (1.81)	6565 (1.48)	9692 (2.12)
Asian	1967 (0.52)	2528 (0.57)	5190 (1.17)	2502 (0.57)
Indigenous	495 (0.13)	612 (0.14)	607 (0.14)	605 (0.14)
Native Hawaiian or Pacific Islander	204 (0.05)	246 (0.06)	248 (0.06)	252 (0.06)
Multiracial	16 (0.01)	24 (0.01)	24 (0.01)	22 (0.01)
Other	87,448 (23.1)	108,777 (24.5)	108,385 (24.5)	108,017 (24.4)

a MICE: multiple imputation using chained equations.

b NH: notHispanicc.

**Table 5.** Likelihood ratio test results comparing models with and without race and ethnicity as predictors of flu vaccination. Race and ethnicity were imputed in the West Virginia Statewide Immunization Information System using data collected between 2021 and 2022. Models were fit to datasets imputed using Stata’s multiple imputation using chained equations (based on Rubin’s rules), Iterative-Imputer (Python scikit-learn), and Miceforest (Python).

Variable and method	G-Statistic	*P* value	Degrees of freedom	Between-Imputation Variance^a^
Race				
Stata MICE^b^	26,452.659	<.001	—^c^	4103.382
Iterative-Imputer	128,280.274	<.001	6	—
Miceforest	26,891.527	<.001	6	—
Ethnicity				
Stata MICE	1142.231	<.001	—	1277.885
Iterative-Imputer	1.683	.19	1	—
Miceforest	2185.012	<.001	1	—

a Between-imputation variance is only applicable to Stata’s MICE model.

b MICE: multiple imputation using chained equations

c Not applicable.

### Iterative-Imputer

347,633 race categories that were previously missing were successfully imputed after completing the Iterative-Imputer. There were 0.26% additional White, 142.5% additional Black, 50.4% additional Asian, 0% additional Indigenous, 95.2% additional Native Hawaiian or Pacific Islander records, 10.6% additional Multiracial, and 0% additional Other records after imputations (Table 1).

Like MICE, 780,339 ethnicity categories that were previously missing were imputed after completing Iterative-Impute r. There were 0% additional Hispanic or Latino and 4.5% Not Hispanic or Latino records after imputations (Table 1).

After the Iterative Imputer, individual demographics did not remain proportional to the original dataset distributions (Figures1  2 and Table 2). A chi-square test illustrated that there was a statistically significant difference between the complete case and the Iterative-Imputer estimation (*P*<.001; Table 3). Overall computational time was 2 minutes.

For the 2021‐2022 flu season, the complete case analysis flu vaccine coverage rate was 26%. Using Iterative-Imputer, after imputations, West Virginia had an overall flu vaccine coverage rate of 19%. Flu vaccine coverage rates decreased when stratified by race and ethnicity when compared with a complete case analysis, with the most significant decreases observed in NH White (3%), NH Black (13%), Native Hawaiian or Pacific Islander (4%), and other (15%; Figure 3 and Table 4). After reconciling missing race and ethnicity, an additional 63,984 individuals were included in the analysis that were previously excluded from stratified analyses.

Likelihood ratio tests were used to determine the impact of race and ethnicity on vaccine uptake. Applying Iterative Imputer, race was significantly associated with flu vaccination status (G-statistic=128,280.27, *P*<.001), demonstrating a massive effect (Table 5). For ethnicity, the Iterative-Imputer model produced a nonsignificant result (G-statistic=1.68, *P*=.195; Table 5).

### Miceforest

347,633 race categories that were previously missing were imputed after completing Miceforest. There were 16.5% additional White records, 17.9% additional Black records, 15.9% additional Asian records, 27.7% additional Indigenous records, 26.0% additional Native Hawaiian or Pacific Islander records, 13.6% additional Multiracial records, and 15.1% additional Other records after imputations (Table 1).

780,339 ethnicity categories that were previously missing were imputed after completing Miceforest. There were 76.1% additional Hispanic or Latino records and 39.8% additional Not Hispanic or Latino records after imputations (Table 1).

After Miceforest, individual demographics remained proportional to the original dataset distributions (Figures1  2 and Table 2). A chi-square test illustrated that there was a statistically significant difference between the complete case and Miceforest estimates (*P*<.001; Table 3). Overall computational time was 10 minutes.

For the 2021‐2022 flu season, the complete case analysis flu vaccine coverage rate was 26%. Using miceforest, after imputations, West Virginia had an overall flu vaccine coverage rate of 19%. Flu vaccine coverage rates decreased when stratified by race and ethnicity when compared with a complete case analysis, with the most significant decreases observed in NH White (5%), NH Black (4%), Hispanic or Latino (7%), Asian (7%), Native Hawaiian or Pacific Islander (7%), and Other (18%; Figure 2 and Table 4). After reconciling missing race and ethnicity, an additional 63,984 individuals were included in the analysis that were previously excluded from stratified analyses.

Likelihood ratio tests were used to determine the impact of race and ethnicity on vaccine uptake. Using Miceforest, race was significantly associated with flu vaccination status (G-statistic=26,891.53; *P*<.001), demonstrating a large effect (Table 5). For ethnicity, the Miceforest model remained robust and was significantly associated with flu vaccination status (G-statistic=2185.01; *P*<.001; Table 5).

## Discussion

### Principal Findings

Our results highlight that the imputation method can profoundly change research findings. For race, all 3 methods show highly significant results and conclude that there are significant race disparities in flu vaccination after addressing missing data. G-tests performed on race and ethnicity and flu vaccination contingency tables showed consistent evidence of significant racial disparities across all imputation methods (*P*<.001). However, for ethnicity, Iterative-Imputer failed to distinguish any significant disproportions, while MICE and Miceforest found strong associations.

Of the 3 methods used to address missing data, we found that MICE and Miceforest had the best model fit and effect sizes. MICE and Miceforest were able to reconcile missing data with little bias, produced more stable and reliable imputations, and retained better preservation of original data relationships, producing similar conclusions; subsequently allowing for more sophisticated post hoc analyses. Iterative Imputer exhibited the most substantial deviations from the complete case analysis. While the majority of race and ethnicity categories retained proportions similar to those observed before imputation, the Black race category had a notable 142% increase post-Iterative-Imputer. Though all methods identify disparities for race, Iterative-Imputer suggests effects that are 5 times stronger than MICE and Miceforest (Iterative-Imputer G-statistic=128,280; MICE G-statistic=26,453; Miceforest G-statistic=26,892). Alternatively, Iterative-Imputer suggests no significant (*P*=.19) ethnicity disparities, while MICE and Miceforest distinguish strong disparities. Iterative-Imputer’s results suggest that the model may be overfitting that data, intensifying present correlations, or generating false patterns in the imputed dataset. The significant change within a singular racial and ethnic group carries several potential implications: it may lead to neglecting crucial health disparities or greatly overvaluing disparities.

While MICE and Miceforest demonstrated more reliable and moderate effect sizes, the most striking difference between MICE and Miceforest was overall computational time. Large population surveillance-based datasets can be computationally expensive to impute [50]. While traditional MICE and Miceforest were proportionally similar to the complete case analysis and had similar G-statistics, the computational load was a significant limitation for further epidemiological analyses. Since Stata 17 operates locally, executing MICE required approximately 14 hours, using between 92% and 98% of the total central processing unit (CPU) capacity of the local system. In contrast, as Iterative-Imputer and Miceforest were processed on a cloud-based drive, the total computational time was reduced to under 10 minutes. Cloud-based computing extends several advantages, including enhanced scalability, reduced dependence on local servers, and the capability to allocate computational jobs efficiently [51,52]. As datasets become larger and more complex, they require larger memory and CPUs become necessary, and consequently, there will be computational limitations. By divesting resource-intensive tasks to the cloud, researchers and public health officials can alleviate processing constraints, enabling analogous performance of multiple tasks and minimizing downtime. This improved efficiency not only accelerates analyses but also improves overall productivity by removing lengthy processing delays.

Compared with the complete case analysis, the imputed dataset was 45% larger, and the estimated 2021‐2022 flu vaccination coverage rate decreased from 26% to 19%. This resulted in distinct divergences in vaccination rates from those seen in the complete case analysis. Stratified flu vaccination coverage rates declined across all racial and ethnic categories following imputation. This is both expected, due to denominator inflation, and consistent with existing literature, which suggests that enhancements in data completeness often uncover underlying racial inequities [2,6-8,10,14,53].

In addition, the presence of the “Other” category may be artificially influencing the race missingness rate. Reports indicate that the increasing size of the “Other” category may be influenced by individuals choosing not to disclose their race, varying cultural perceptions of race, or vaccine providers selecting “Other” rather than leaving the race field blank [54,55]. Those who select the “Other” category get combined into a single, dissimilar category that does not reflect accurate identities and makes results for this group difficult to accurately decipher.

These findings align with the broader imputation literature, which demonstrates that not only does reconciling missing race and ethnicity data reveal under-reported disparities, but that imputation methodology can significantly influence the magnitude of these inequities. Similar patterns have been observed in findings from Labgold et al [2], Dorabawila et al [42], and Russ et al [41], where imputing missing race and ethnicity revealed greater disparities in health outcomes than previously understood, but the severity of these inequities fluctuated contingent upon parameters placed on the imputation method. This is consistent with theoretical bases proposing that the creation and assumptions of statistical models and algorithms can either dilute or intensify disparities, conditional on how missingness is managed and whose data are omitted [2,30,39,56]. The absence of demographic data in IISs carries significant public health implications. Missing data limits our capability to correctly assess and monitor vaccination coverage, potentially leading to an incomplete or inaccurate understanding of disparities in vaccination rates among different vulnerable groups. Consequently, public health interventions and policies may be based on flawed assumptions, undermining their effectiveness. Moreover, the inability to identify and address disparities hinders efforts to promote equity within communities. Poor data quality can also result in inefficient allocation of resources, both human and financial, further exacerbating inequities. Finally, inadequate data can erode public trust in health systems, as decisions based on incomplete information may be perceived as unreliable or inequitable. However, Miceforest successfully imputed missing values in a large public health dataset in the most time-efficient manner, and therefore, we were able to mitigate potential bias and increase our statistical power in our analyses. By allowing more complete and representative datasets, Miceforest encourages more equitable vaccination surveillance and permits fuller public health decision-making in a timely manner. State public health departments can use this technique to augment incomplete data to identify vaccine coverage gaps more accurately, concentrate on programming and resource allocation in underserved communities, and assess whether interventions are aiding populations who need it most. Our findings emphasize how multiple imputation is not only a statistical solution but a means for progressing equity in immunization programs and interventions.

There were several limitations to our approach. Due to high levels of missingness of variables in the dataset, there were limited informative variables for the imputations. The WVSIIS population denominator is higher than the census population, which can skew flu vaccination rates lower than they are. Race and ethnicity are self-reported, and they may not reflect the reality of demographic distribution in the state, especially with growing nuances of racial and ethnic identity.

Data are the foundation of all effective public health interventions, and missing data can reduce our comprehension of vaccination coverage, recognizing disparities, and creating successful public health programs. Without properly addressing missing data, vulnerable groups continue to be underserved. As demonstrated, different methods for reconciling missing data result in different assumptions regarding the data and the process. However, using Miceforest to reconcile missing demographic data poses a potential solution, offering a flexible, fast, and iterative approach that can improve data completeness while preserving underlying distributions and mitigating potential bias.

Future efforts should validate imputation values against other population-based surveillance systems, such as census data and claims data. In addition, because our imputations focused on one state’s IIS, supplementary efforts should examine the performance of various multiple imputation methods across more diverse populations and geographies and examine the optimal frequency for updating imputation models. Further, based on our results, we recommend several suggestions for enhancing both the epidemiological approach and practice in immunization surveillance. Though more resource-intensive, preventing missing data initially is the most effective measure to handle missing data, especially for surveillance data that necessitates system-wide resolutions. This can be achieved through standardized race and ethnicity data collection protocols and data collection training in state IISs. Imputation should be a temporary answer for a larger, systematic problem. Public health agencies should be transparent in the imputation methods used in reports and present both complete case and imputed estimates to demonstrate the impact of missing data on vaccine equity reports.

### Conclusions

Both MICE and Miceforest offer flexible and reliable approaches for imputing missing demographic data in IISs, outperforming Iterative-Imputer with regard to bias mitigation and distributional accuracy. These findings highlight that imputation method selection can greatly affect research outcomes, with repercussions for both statistical validity and public health decision-making and trust. While MICE and Miceforest yielded comparable effect sizes and preserved demographic proportions, MICE’s substantial computational demands limit its scalability for large datasets, while Miceforest’s ability to leverage cloud-based computing enhances efficiency by offloading resource-intensive tasks, enabling parallel execution, and minimizing processing delays.

Addressing missing data is both a methodological necessity and a public health imperative when handling large surveillance data. By improving data completeness and analytical precision, multiple imputation methods such as Miceforest can help illuminate disparities, reveal disparities, inform resource allocation, and promote equity in immunization and public health programs.

## Abbreviations

ATSDR: Agency for Toxic Substances and Disease Registry
BISG: Bayesian Improved Surname and Geocoding
CDC: Centers for Disease Control and Prevention
CPU: central processing unit
FORHP: Federal Office of Rural Healthy Policy
IIS: immunization information systems
IRB: institutional review board
MICE: multiple imputation using chained equations
ML: machine learning
NH: non-Hispanic
SVI: Social Vulnerability Index
WVSIIS: West Virginia Statewide Immunization Information System
